# Supplementary material for: Sports and Energy Drink Consumption, Oral Health Problems and Performance Impact among Elite Athletes
Source: Nutrients. 2022 Nov 30;14(23):5089. doi: 10.3390/nu14235089 (PMC9738880; doi:10.3390/nu14235089)
Supplement: Supplementary file 1 [file nutrients-14-05089-s001.zip › Supplementry file S1.pdf]

KHYBER MEDICAL UNIVERSITY PESHAWAR

IBMS INSTITUTE OF BASIC MEDICAL SCIENCES

DATA COLLECTION QUESTIONNAIRE

Title of the study: Oral Health Status, Behaviors and Nutrition in Elite Pakistani Athletes

پاکستانی ایتھلیٹس میں منہ اور دانتوں کی صحت، عادات اور غذائیت

Participant code ☐ ☐ ☐ ☐

شرکت کرنے والے کا کوڈ

Sports and Energy Drinks Consumption Questionnaire

توانائی اور کھیلوں کے مشروبات سے متعلق سوالنامہ

1. Do you or did you ever used any of the following products, at least once a week in the past 12 months?

کیا آپ نے پچھلے ایک سال کے دوران مندرجہ ذیل میں سے کوئی بھی پروڈکٹ ہفتے میں ایک مرتبہ استعمال کی ہے؟

☐ Sports & Energy Drinks----- توانائی اور کھیلوں کے مشروبات
☐ Energy Bars------ توانائی بار
☐ Energy gels -----توانائی جیل
☐ Never used any--- کبھی استعمال نہیں کی ہے

(If no, stop the interview at this point) اگر کبھی استعمال نہیں کی ہے تو یہاں پر انٹرویو ختم کریں

2. If yes, write brand name(s) please.---اگر ہاں تو برائے مہربانی برانڈ نام درج کریں

Sports & Energy drinks: ------------------------------------- -------------------------------- -------------------------------

Energy Bars: ------------------------------------- -------------------------------- ------------------------------

Energy Gels: ------------------------------------- -------------------------------- -------------------------------

3. How many times, do you consume these products normally? Please tick one.عمومی طور پر آپ کتنی مرتبہ یہ پراڈکٹ استعمال کرتے ہیں

☐ 1-2 times per week ----- ہفتے میں ایک سے دو مرتبہ
☐ 3 – 6 times per week--- ہفتے میں تین سے 6 مرتبہ
☐ One time per day-----دن میں ایک بار
☐ two times per day----دن میں دو بار
☐ 3 or more times per day----- دن میں تین یا تین سے زیادہ بار

4. When do you usually consume these products? Multiple answers possible آپ کس وقت یہ پراڈکٹ استعمال کرتے ہیں

☐ Before training---ٹریننگ سے پہلے
☐ During training---ٹریننگ کے دوران
☐ After training --ٹریننگ کے بعد
☐ Before competition--مقابلے سے پہلے
☐ During competition---مقابلے کے دوران
☐ After competition---مقابلے کے بعد

5. Reasons for using these products? Multiple answers possible استعمال کے وجوہات--ایک سے زیادہ جوابات ممکن ہے

☐ Provides energy--- توانائی فراہم کرتا ہے
☐ Replenish lost energy---توانائی بحال کرتا ہے
☐ Replaces body fluids loss---سیال مادہ بحال کرتا ہے
☐ Reduce fatigue ------تھکن کم کرتا ہے
☐ Improve performance ---کارکردگی بہتر بنانے کے لئے
☐ Reduce stress ----ذہنی تناؤ کم کرنے کے لئے
☐ Other specify----دیگر وضاحت کریں -----
