# Supplementary material for: Sports and Energy Drink Consumption, Oral Health Problems and Performance Impact among Elite Athletes
Source: Nutrients. 2022 Nov 30;14(23):5089. doi: 10.3390/nu14235089 (PMC9738880; doi:10.3390/nu14235089)
Supplement: Supplementary file 1 [file nutrients-14-05089-s001.zip › Supplementry file S2.pdf]

KHYBER MEDICAL UNIVERSITY PESHAWAR

IBMS INSTITUTE OF BASIC MEDICAL SCIENCES

QUESTIONNAIRE سوالنامہ

Title: Oral Health Status, Behaviors and Nutrition in Elite Pakistani Athletes

پاکستانی ایتھلیٹس میں منہ اور دانتوں کی صحت‘عادات اور غذائیت

**Section 1: General Information** سیکشن 1 عمومی معلومات			
**A1**:Participant code<br>شرکت کرنے والے کا کوڈ	☐ ☐ ☐ ☐	**A2**: Participant name<br>شرکت کرنے والے کا نام	
**A3**: Phone Number<br>فون نمبر		**A4**: Interview Date<br>انٹرویو تاریخ	
**A5**: Gender<br>جنس	☐ Male -------- مرد<br>☐ Female ------- عورت	**A6**: Age (in years)<br>عمر سالوں میں	
**A7**: What is your mother tongue?<br>آپ کی مادری زبان کیا ہے؟	☐ Pashto ------(پشتو)<br>☐ Punjabi------ پنجابی	☐ Hindko----------- ہندکو<br>☐ Other specify----دیگر وضاحت کریں	
**A8**: What is your marital status?<br>آپ کی ازدواجی حیثیت کیا ہے؟	☐ Single-------------غیر شادی شدہ<br>☐ Married--------------شادی شدہ -<br>☐ Other specify-----دیگر وضاحت کریں		
**A9**: What is the main sports you are playing?<br>آپ کون سا کھیل کھیلتے ہیں؟			
**A10**: What is your athletic caliber?<br>آپ کس سطح پر کھیلتے ہیں؟	☐ International --------- بین الاقوامی<br>☐ National---------------- قومی<br>☐ College, University-----کالج یا یونیورسٹی<br>☐ Other specify-----دیگر وضاحت کریں		
**A11:** How many hours do you train per week? (Approximately)<br>آپ ہفتے میں تقریبا کتنا ٹریننگ لیتے ہیں؟			
**A12:** What is your level of education attained?<br>آپ نے کتنی تعلیم حاصل کی ہے؟			
**A13:** Do you have any formal nutrition study? E.g course/diploma/certificate?<br>آپ نے نیوٹریشن کے متعلق کوئی کورس لیا ہے مثلاً ڈگری سرٹیفیکیٹ ڈپلومہ وغیرہ	☐ Yes---------- ہاں<br>☐ No-----------نہیں		
**A14:** What is your monthly household income? From all sources<br>آپ کی ماہانہ آمدن کیا ہے؟			
**A15:** What is your occupation?<br>آپ کا پیشہ کیا ہے؟			

**Section B: Oral health Behaviour/Practices**	منہ اور دانتوں کی صحت اور عادات
**B1:** Do you brush/clean your teeth?<br>کیا آپ دانتوں کی صفائی کرتے ہیں؟	☐ Yes---------- ہاں<br>☐ No-----------نہیں
**B2:** How many times, do you brush your teeth every day?<br>آپ دن میں کتنی مرتبہ دانتوں کی صفائی کرتے ہیں؟	☐ One time----- ایک بار<br>☐ Two times------دو بار<br>☐ Three or more time------تین یا زیادہ بار
**B3:** For how long, do you clean your teeth?<br>آپ دانتوں کی صفائی پر کتنا ٹائم لیتے ہیں؟	☐ One or less minute---- ایک یا ایک سے کم منٹ<br>☐ Two minutes--------دو منٹ<br>☐ Three or more minutes---تین یا تین سے زیادہ
**B4:** When do you brush your teeth?<br>آپ کس وقت دانتوں کی صفائی کرتے ہیں؟	☐ In the morning----- صبح<br>☐ Afternoon------- دوپہر<br>☐ Before going to bed---رات سونے سے پہلے<br>☐ Other specify-----دیگر وضاحت کریں
**B5:** What you use for cleaning your teeth?<br>آپ دانتوں کی صفائی کے لیے کیا چیزیں استعمال کرتے ہیں؟	☐ Toothbrush & toothpaste--ٹوتھ برش ٹوتھ پیسٹ<br>☐ Miswak----مسواک<br>☐ Dental floss----- ڈینٹل فلاس<br>☐ Other specify-----دیگر وضاحت کریں
**B6:** Do you use fluoride tooth paste for cleaning teeth?<br>کیا آپ دانتوں کی صفائی کے لیے فلورائڈ ٹوتھ پیسٹ استعمال کرتے ہیں	☐ Yes---------- ہاں<br>☐ No-----------نہیں<br>☐ Don't know----پتا نہیں
**B7:** How often do you replace your toothbrush?<br>کتنا عرصہ بعد اپنا ٹوتھ برش تبدیل کرتے ہیں؟	☐ Three months or less--تین مہینے یا کم<br>☐ 6 month---- 6 مہینے<br>☐ Never until usable---- بالکل نہیں جب تک قابل استعمال ہو
**B8:** How often do you visit dentist?<br>آپ کتنی بار دانتوں کے ڈاکٹر سے ملتے ہیں؟	☐ Regularly every 6-8 months-----باقاعدہ 6-8 مہینے بعد<br>☐ Occasionally-----------------کبھی کبھار<br>☐ When I have dental pain------جب دانت میں درد ہو<br>☐ Never ----------------------کبھی نہیں
**B9:** When did you last visited dentist?<br>آپ آخری بار دانتوں کے ڈاکٹر سے کب ملے تھے؟	☐ Last six month-------------پچھلے 6 مہینے میں<br>☐ Last one year--------------پچھلے سال<br>☐ More than a year---------ایک سال سے زیادہ
**B10:** What was the reason for visiting dentist?<br>دانتوں کے ڈاکٹر سے ملنے کی وجہ؟	☐ Dental pain---------------دانتوں میں درد<br>☐ Family/friend advice-----دوستوں کا مشورہ<br>☐ Doctor/dentist advice----ڈاکٹر کا مشورہ<br>☐ Other specify ---دیگر وضاحت کریں
**B11:** What are the reasons for NOT visiting the dentist?<br>دانتوں کے ڈاکٹر سے نہ ملنے کی وجہ؟	☐ Expensive treatment----علاج کے اخراجات<br>☐ Unavailability of dentist---دانتوں کے ڈاکٹر کا میسر نہ ہونا<br>☐ Other specify ---دیگر وضاحت کریں

Section C: Self-reported oral health	
**C1:** Compared to other people, how would you describe your general health at present?<br>دوسرے لوگوں کے مقابلے میں اپنی صحت کو کس طرح دیکھتے ہیں؟	☐ Very Good---------------- بہت اچھا<br>☐ Good------------------------ اچھا<br>☐ Fair--------------------- اوسط<br>☐ Poor---------------------- خراب<br>☐ Very poor------------- بہت خراب
**C2:** How would you describe your oral health (mouth, teeth and gums) at present?<br>دوسرے لوگوں کے مقابلے میں اپنے منہ اور دانتوں کی صحت کو کیسے دیکھتے ہیں؟	☐ Very Good---------------- بہت اچھا<br>☐ Good------------------------ اچھا<br>☐ Fair--------------------- اوسط<br>☐ Poor---------------------- خراب<br>☐ Very poor------------- بہت خراب
**C3:** Currently or in the past one year, do you have any problem related to your teeth? (Check all that apply)<br>ابھی یا پچھلے ایک سال کے دوران آپ کو دانتوں کا کوئی مسئلہ ہوا ہے؟	☐ Dental pain---------------دانتوں میں درد<br>☐ Sensitivity to hot & cold---دانتوں کو ٹھنڈا گرم لگنا<br>☐ Gum bleeding-----------مسوڑھوں سے خون آنا<br>☐ Bad breath----------منہ سے بدبو آنا<br>☐ Other specify ---دیگر وضاحت کریں
**Section D: Oral health impact on psychosocial life and performance**	**سماجی زندگی اور کارکردگی پر اثرات**
**D1:** Over the past 12 months: Have you had any difficulty eating or drinking because of your mouth, teeth or gums?<br>پچھلے ایک سال کے دوران آپ کو منہ 'دانتوں یا مسوڑھوں میں تکلیف کی وجہ سے کھانے پینے میں مشکل پیش آئی ہے؟‘	☐ Yes---------- ہاں<br>☐ No-----------نہیں
**D2:** Over the past 12 months: Have you had any difficulty relaxing (including sleeping) because of your mouth, teeth or gums?<br>پچھلے ایک سال کے دوران آپ کو منہ دانتوں یا مسوڑھوں میں تکلیف کی وجہ سے آپ کے آرام میں خلل آئی ہے؟‘	☐ Yes---------- ہاں<br>☐ No-----------نہیں
**D3:** Have you had any difficulty smiling, laughing or showing your teeth without embarrassment?<br>کیا آپ کو بغیر شرمندگی کے مسکرانے، ہنسنے یا دانت دکھانے میں کوئی دشواری ہوئی ہے؟	☐ Yes---------- ہاں<br>☐ No-----------نہیں
**D4:** Have you had any difficulties participating in normal training or competition due to problems with your mouth, teeth or gums?<br>آپ کو منہ ' دانتوں یا مسوڑھوں میں تکلیف کی وجہ سے آپ کے ٹریننگ یا مقابلے میں کبھی مشکل پیش آئی ہے؟‘	☐ Full participation without any problem<br>بغیر کسی تکلیف کے ہمیشہ مکمل حصہ لیا ہے<br>☐ Full participation with oral problems<br>ہمیشہ مکمل حصہ لیا ہے لیکن تکلیف کے ساتھ<br>☐ Reduced participation with oral problems<br>دانتوں میں تکلیف کی وجہ سے کم حصہ لیا ہے<br>☐ Could not participate due to oral problems<br>دانتوں میں تکلیف کی وجہ سے بالکل حصہ نہیں لیا ہے
**D5:** To what extent, you reduced your training volume due to mouth, teeth or gum disease, over the last 12 months?<br>آپ نے پچھلے 12 ماہ کے دوران منہ، دانت یا مسوڑھوں کی بیماری کی وجہ سے اپنی تربیت کا حجم کس حد تک کم کیا؟	☐ No reduction-----کمی نہیں کی ہے<br>☐ Reduced------- کمی کی ہے<br>☐ Could not participate at all--بالکل حصہ ہی نہیں لے سکا
